# Case Report: A Novel Truncating Variant of *NR0B1* Presented With X-Linked Late-Onset Adrenal Hypoplasia Congenita With Hypogonadotropic Hypogonadism

**DOI:** 10.3389/fendo.2022.897069

**Published:** 2022-06-16

**Authors:** Feng Zhu, Min Zhou, Xiuling Deng, Yujuan Li, Jing Xiong

**Affiliations:** ^1^ Department of Cardiology, Union Hospital, Tongji Medical College, Huazhong University of Science and Technology, Wuhan, China; ^2^ Department of Pulmonary and Critical Care Medicine, Tongji Hospital, Tongji Medical College, Huazhong University of Science and Technology, Wuhan, China; ^3^ Key Laboratory of Respiratory Diseases, National Ministry of Health of the People’s Republic of China and National Clinical Research Center for Respiratory Disease, Wuhan, China; ^4^ Department of Endocrinology, Union Hospital, Tongji Medical College, Huazhong University of Science and Technology, Wuhan, China; ^5^ Department of Internal Medicine, Distinct HealthCare, Wuhan, China; ^6^ Department of Nephrology, Union Hospital, Tongji Medical College, Huazhong University of Science and Technology, Wuhan, China

**Keywords:** adrenal hypoplasia congenita, hypogonadotropic hypogonadism, NR0B1 gene, DAX1, X-linked recessive

## Abstract

Nuclear receptor subfamily 0 group B member 1 gene (*NR0B1*) encodes an orphan nuclear receptor that plays a critical role in the development and regulation of the adrenal gland and hypothalamic–pituitary–gonadal axis. In this study, we report a novel mutation in *NR0B1* that led to adult-onset adrenal hypoplasia congenita (AHC) and pubertal development failure in a male adult. Clinical examinations revealed hyponatremia, elevated adrenocorticotropic hormone levels, reduced testosterone and gonadotropin levels, and hyper-responses to gonadotropin-releasing hormone and human chorionic gonadotropin stimulation tests. Whole-exome sequencing and Sanger sequencing were performed to identify the potential causes of AHC. Candidate variants were shortlisted based on the X-linked recessive models. Sequence analyses identified a novel hemizygous variant of c.1034delC in exon 1 of *NR0B1* at Xp21.2, resulting in a frameshift mutation and premature stop codon formation. The c.1034delC/p.Pro345Argfs*27 in the *NR0B1* gene was detected in the hemizygous state in affected males and in the heterozygous state in healthy female family carriers. These results expand the clinical features of AHC as well as the mutation profile of the causative gene *NR0B1*. Further studies are needed to elucidate the biological effects of the mutation on the development and function of the adrenal gland and the hypothalamic–pituitary–gonadal axis.

## Introduction

The nuclear receptor subfamily 0 group B member 1 (*NR0B1)* gene encodes DAX1, an orphan nuclear receptor that plays a critical role in the development and differentiation of the adrenal gland and hypothalamus-pituitary-gonadal axis (1). It is predominantly expressed in the adrenal cortex, hypothalamus, pituitary gland, and gonads (testis and ovary). DAX1 has been proposed to serve as a transcription factor involved in the development of the adrenal cortex and pituitary gonadotropes (2). More than 200 mutations have been reported in *NR0B1*, most of which are nonsense or frameshift mutations that result in premature truncation of the protein (3). Defective *NR0B1* leads to X-linked adrenal hypoplasia congenita (AHC), resulting in a failure to develop the permanent adult adrenal cortex (4). Patients with *NR0B1* mutations may present with severe salt wasting in infancy or have a more insidious onset during childhood. AHC rarely presents with adrenal failure later in adulthood, and late-onset primary adrenal insufficiency (AI) is often accompanied by hypogonadotropic hypogonadism (HH) and azoospermia (5–7). In this study, we report a novel mutation in *NR0B1* that leads to late-onset AHC and failure of pubertal development.

## Materials and Methods

### Subjects

**Figure 1:**
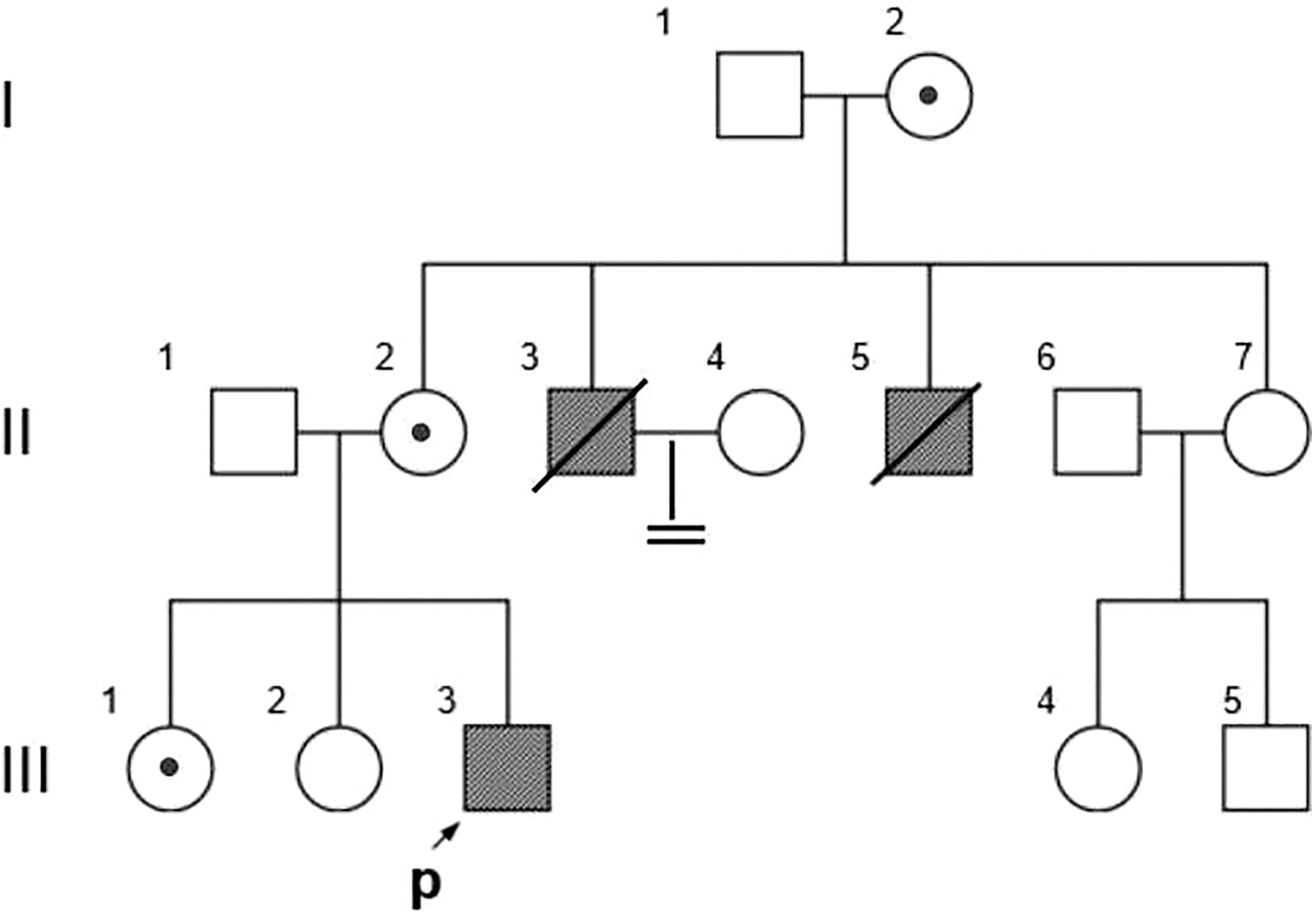
Family Pedigree. Symbols represent males (squares), females (circles), affected subjects (solid symbols), carriers (dotted symbols), and the proband (arrow). The propositus is indicated by an arrow.

The proband and all available family members were recruited from Wuhan Union Hospital (**Figure 1**). Medication history, disease symptoms, and progression of each family member was recorded by dictation as well as from medical records. Venous blood samples were collected from each family member after obtaining written informed consent. The present study was approved by the ethics committee of Wuhan Union Hospital.

### DNA Sequencing

A library of genomic DNA was created from the proband’s blood sample and enriched for exon targets according to the protocols of the Agilent SureSelect Human All Exon V6 Kit. After validation for size distribution using the Agilent 2100 Bioanalyzer, the enriched library was sequenced on the Illumina HiSeq X Ten platform. Raw files were generated and mapped to the human reference genome (GRCh37/hg19) using the Burrows-Wheeler alignment tool (version 0.7.8-r455). SAMtools was then applied to call single-nucleotide variants (SNVs) and indels (deletions and insertions, < 50 bp). Subsequently, ANNOVAR, accompanied by several prediction tools, was used to annotate SNVs and indels. Variants were annotated using genomic coordinates, referent nucleotides, variant nucleotides, mutation types, alleles, allele frequencies, gene names, amino acids variants and their evolutionary conservation. For allele frequency, each variant was compared against public population genetic databases. Based on the variant annotations, we focused on nonsynonymous coding substitutions, frameshifts, and splicing site mutations on chromosome X. Post these filtering steps, a list of candidate variants and related genes was created. To prioritize the most likely candidate disease-causing genes, all candidate genes were ranked using Phenolyzer (8). To validate the suspected variant in the proband, PCR and Sanger sequencing were performed on DNA samples of all available family members (II-2, II-3, II-6, III-1, III-2, III-3, III-4, and III-5) (**Figure 1**). The primer pairs used for PCR amplification of the region encompassing the suspected variant were forward 5′-GCTTTTAAAGAGCACCCGCC-3′ and reverse 5′-TTTCTTCACCTTTGCCCCGAC-3′.

## Case Presentation

### Clinical Presentations

**Figure 2:**
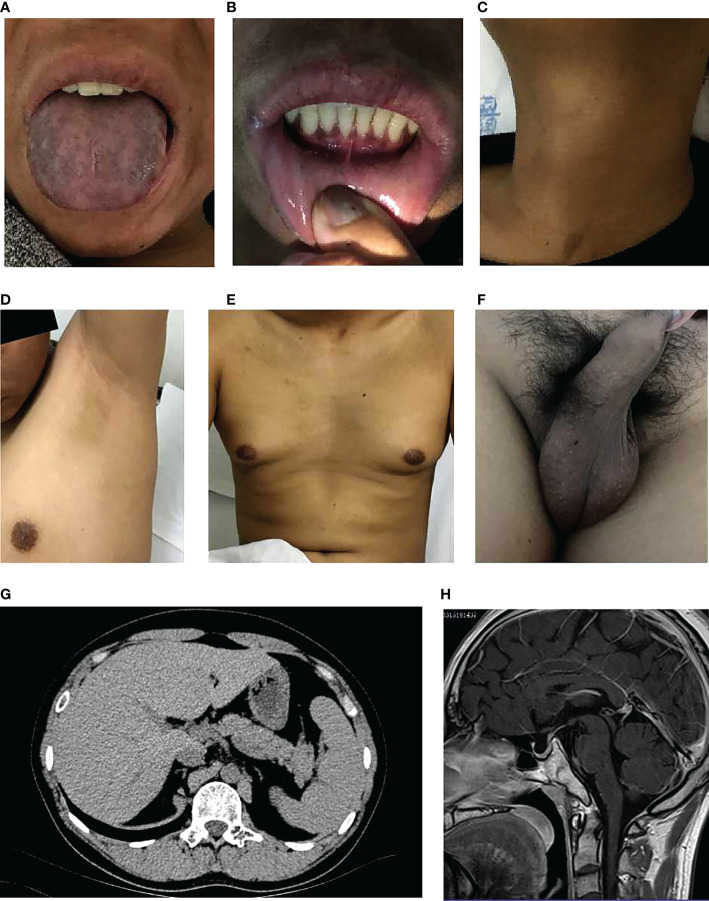
Clinical characteristics of the proband. **(A)** Significant melanosis could be seen in the wrinkles of the face, gums, tongue, and limbs. **(B)** A few hyperpigmented macules were noted on the lips and oral mucosa. **(C-F)** Inconspicuous male secondary sexual characteristics: no beard, axillary hair, no obvious laryngeal knot, inverted triangle distributed pubic hair, significantly reduced bilateral testicles. **(G, H)**. Scanning results (adrenal hypoplasia, hypogonadotropic hypogonadism, small bilateral testes, olfactory sulci and pituitary, mild fatty liver).

The pedigree in **Figure 1** shows the inheritance pattern of the X-linked recessive disorder. The proband (III-3), a 26-year-old male patient (height: 5 feet; weight: 75 kg; BMI 25.95), was admitted to the hospital with suspected adrenocortical failure. Medical history revealed that the patient had not exhibited secondary sexual characteristics by 17 years of age and had initial symptoms of skin hyperpigmentation; he had initiated prednisone oral tablet 5 mg per day along with some unknown Chinese herbs to help grow pubic hair. Up until the age of 24 years, there had been no incidence of fatigue, weight loss, dehydration, anorexia, nausea, or vomiting; however, in the following 2 years, he had developed worsening skin hyperpigmentation and experienced nausea and weakness following occasional treatment interruptions. Physical examination revealed generalized hyperpigmentation (**Figures 2A, B**) and delayed puberty, with the absence of secondary sexual characteristics (**Figures 2C–F**).

Laboratory examinations revealed a serum sodium level of 123.6 mmol/L at admission, which confirmed hyponatremia; however, it elevated to 136.6 mmol/L following hydrocortisone treatment. The serum potassium levels were normal. The serum triglyceride level was extraordinarily high (29.57 mmol/L) at admission but dropped to 3.91 mmol/L following 5 days of lipid-lowering treatment. The serum cholesterol level was high (7.18 mmol/L). The plasma cortisol level (11.017 μg/L) was low. The adrenocorticotropic hormone level was extremely high, above the upper limit of detection (> 2000 pg/ml), which was consistent with primary AI. The VLCFA (very long-chain fatty acids) and 17-Hydroxyprogesterone concentrations were normal (**Table 1**). Initial hormone examinations showed normal levels of luteinizing hormone (2.49 IU/L) and follicle-stimulating hormone (3.08 IU/L) but a low level of testosterone (2.14 nmol/L). Thereafter, both gonadotropin-releasing hormone (GnRH) and human chorionic gonadotropin (hCG) stimulation tests were performed to diagnose the cause of hypotestosteronemia. As depicted in **Table 2**, in response to GnRH, testosterone levels increased from 1.96 IU/I to 10.27 IU/I at 90 s, but then decreased to 9.67 IU/I at 120 s. In response to hCG, testosterone levels increased by more than nine-fold, from 2.14 nmol/L to 18.33 nmol/L, after 48 h of stimulation, indicating that the testicular tissue was capable of achieving normal secretory function upon stimulation.

**Table 1:** Basal biochemical and hormone measurements.

Measures	At admission	After treatment	Normal value
Na/K	123.6/4.3	136.6/4.5	[135–150]/[3.5–5.5] Eq/L
ACTH	>2000	NA	[9–40] pg/mL
Cortisol	11.017	NA	[37–194] μg/L
Triglyceride	29.57	5.59	[<1.7] mmol/L
Cholesterol	7.18	5.28	[2.9-6.0] mmol/L
17-OHG	2.5	NA	[0.7–3.6ng/mL] ng/mL
VLCFA	0.30	NA	[<0.50] μg/ml
LH	2.49	NA	[1.7–8.6] IU/L
FSH	3.08	NA	[1.5-12.4] IU/L
Testosterone	2.14	NA	[8.6-29.0] nmol/L
Glucose	6.0	NA	[3.9-6.1] mmol/L

NA, not available.

**Table 2:** GnRH stimulation test and hCG stimulation test.

GnRH	
Time	LH(IU/L)
0’	1.96
30’	6.85
60’	9.95
90’	10.27
120’	9.67
**hCG**	
**Time**	**Testosterone(nmol/L)**
0 hr	2.14
48 hr	18.33
72 hr	19.55

The scanning results provided additional evidence of adrenal hypoplasia and HH. Chromosomal analysis confirmed a normal male 46, XY karyotype. Bilateral testicular volume reduction was detected using B-ultrasound (left: 25.7*19.4*12.7 mm, right: 27.4*18.2*12.8 mm). Abdominal computed tomography revealed bilateral adrenal gland hypoplasia (**Figure 2G**). Magnetic resonance imaging of the olfactory sulci and pituitary gland was normal (**Figure 2H**). In addition, ultrasonography of the upper abdomen showed a mildly fatty liver.

Steroid supplementation with hydrocortisone (15-35 mg/d) and fludrocortisone (100 μg/d) resulted in a rapid improvement in the clinical conditions of the proband; the laboratory results following treatment are listed in **Table 1**. For personal reasons, in addition to androgen replacement, patient refused other treatments such as spermatogenesis therapy. During the follow-up, the patient refused to discuss his condition. His sibling mentioned that the proband’s uncle died in a car accident, and the proband himself was bitter regarding not being able to have children, causing his depression and gloominess. He had abandoned his family plans.

### Mutation Detection

**Figure 3:**
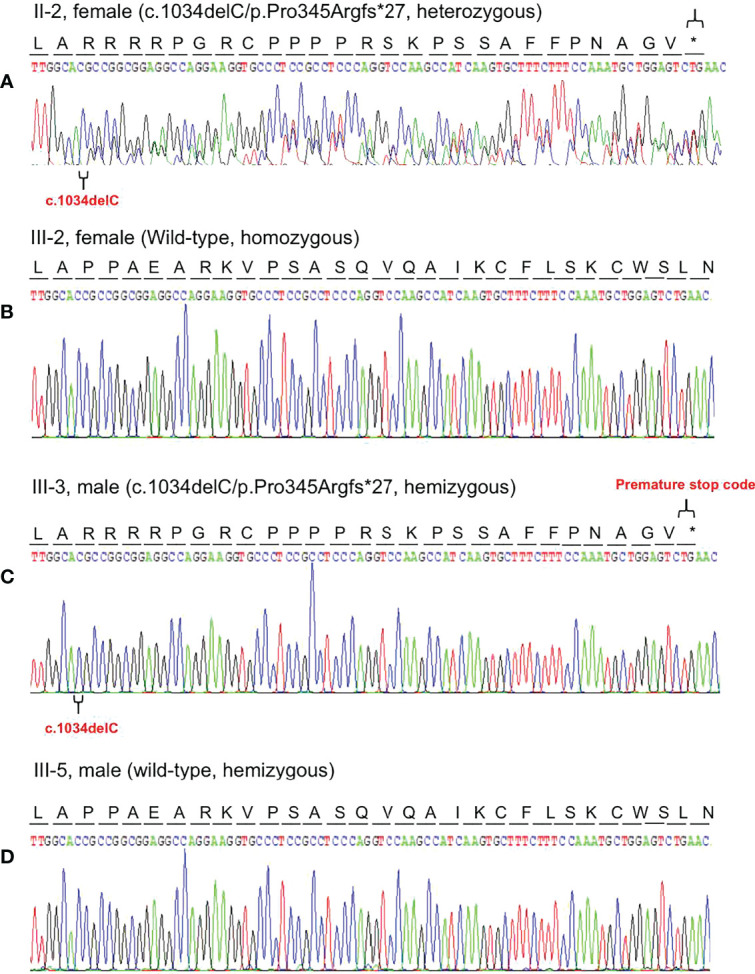
Representative chromatogram of the *NR0B1* gene. The position of the mutation is indicated by special symbols (“
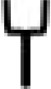
” for the mutation, and “*” for Premature stop code). **(A)** The proband’s mother (II-2) inherited the c.1034delC variant in heterozygous status. **(B)** His health sister (III-2) presented with homozygous wild-type of the *NR0B1* gene. **(C)** The proband was hemizygous for the c.1034delC variant in the *NROB1* gene. **(D)** His maternal cousin (III-5) showed hemizygosity for wild-type of the *NROB1*gene.

Based on the reads aligned to the human reference genome (GRCh37/hg19), a total of 65,535 variants, including 58,704 SNVs and 6,831 indels, were identified. There were 965 variants located on chromosome X. Among them, 167 were nonsynonymous coding substitutions, and eight were frameshift variants. Prioritization with phenotype “adrenal hypoplasia” in Phenolyzer revealed a novel hemizygous frameshift deletion c.1034delC in the first exon of *NR0B1* at Xp21.2. A cytosine deletion at c.1034 caused a frameshift change, with proline-345 being the first affected amino acid by becoming arginine and forming a new reading frame resulting in a premature stop codon 27 amino acids further down (**Figure 3**). As a result, the putative ligand-binding domain (LBD) of the DAX protein was 20 amino acids shorter than the wild type. To validate the suspected variant in the proband, PCR and Sanger sequencing were performed on DNA samples of all available family members (II-2, II-3, II-6, III-1, III-2, III-3, III-4, and III-5), which revealed a hemizygous state in affected males (II-3 and III-3) and a heterozygous state in healthy female carriers (II-2 and III-1) (**Figure 1**).

## Discussion

X-linked AHC is an X-linked recessive inherited disease that typically affects males and presents as primary AI in early infancy or childhood, and HH at puberty with impaired spermatogenesis. Approximately 60% of male patients with AHC develop AI within 2 months after birth, whereas 40% do so between the age of 1 to 9 years. Recently, various cases of late-onset forms of X-linked AHC have been reported. In this study, we report a case of late-onset X-linked AHC and HH caused by a novel variant of *NROB1*. The proband presented with AI at 17 years of age and a lack of development of secondary sexual characteristics during puberty. Sequencing of *NR0B1* revealed a c.1034delC frameshift variant in two affected males in the kindred. The diagnosis of X-linked AHC and HH was established based on the clinical presentation, laboratory tests, and molecular analysis. According to the family history, two other male members of the family had presented with similar manifestations; one (II-3) was diagnosed with Addison’s disease (AD) and male infertility at the age of 30 years and had been treated with prednisone, whereas the other (II-5), who had also been diagnosed with AD at the age of 33 years, succumbed to adrenal crisis at the age of 35 years. This indicates an X-linked mode of inheritance, as was confirmed by the results of the genetic analysis presented herein. In addition, no evident secondary causes of AD were identified, thus allowing us to exclude its presence.

*NR0B1* consists of two exons and spans a 4.96 kb region at Xp21.2. It encodes a 470-amino-acid member of the nuclear hormone receptor superfamily. Most of the coding sequence is found in exon one (9, 10), which encodes the N-terminal domain and part of the C-terminal domain of the protein *NR0B1*. Exon two encodes the remaining part of the C-terminal domain. According to the statistical data of the Human Gene Mutation Database (http://www.hgmd.cf.ac.uk/ac/index.php, accessed on 20180630), a total of 252 mutations have been found in *NR0B1*. Truncating mutations such as nonsense or frameshift mutations have been identified throughout the gene. In contrast, missense changes have tended to cluster in the carboxyl terminus of the protein, corresponding to the putative LBD. Several cases of changes in the domain such as p.Ser259Pro, p.Pro279Leu, p.Ile439Ser, p.Tyr380Asp, and p.Gln305Hisfs*67 have been associated with a milder or late-onset phenotype (11). In functional studies of Tyr380Asp and Ile439Ser mutants, the partial loss of *NR0B1* transcriptional repression was consistent with the late-onset phenotype (12). The p.Pro345Argfs*27 mutation is a novel frameshift mutation within the LBD that introduces premature termination of the *NR0B1* protein, thus resulting in a truncated one. All the affected males in the family exhibited a late-onset phenotype. These findings suggest that the truncated *NR0B1* protein produced by this mutation may retain part of its function. Further studies are needed to elucidate the function of p.Pro345Argfs*27.

The same variant was identified in another male family member (II-5), who had presented with a dysfunction of the adrenal cortex and had deceased owing to adrenal crisis. Although the dysfunction of his gonadal axis had occurred at an older age compared to that of the proband, it was more severe. His impairment involved the hypothalamus and pituitary, whereas the impairment of the proband primarily involved the hypothalamus. This suggests that, despite possessing the same genetic variant, there are differences in clinical manifestations, even in patients of the same pedigree. Liu also reported a nonsense variant change in exon 1 (c.192C>G) in two male siblings presenting with precocious puberty and late-onset HH but exhibiting different clinical manifestations (13).

There was no evident correlation between the *NR0B1* variant type and the clinical presentation. Literature search revealed 11 reports of adult-onset AHC (6, 7, 11, 14–20). The reported cases of adult-onset AI and hypogonadotrophic hypogonadism associated with mutations of *NR0B1* gene are summarized in **Supplementary Table S1**. Of the 11 reports, three (p.Gln37X in one, p.Trp39X in two kindreds) are amino-terminal nonsense mutations, whereas eight (p.Tyr378Cys, p.Leu386Phe, p.Ser259Pro in two kindreds, p.Pro279Leu, p.Gln305Hisfs*67 (c.915delG), p.Tyr380Asp, p.Ile439Ser) are mutations distributed throughout the carboxyl LBD. The mutation described in this study also falls in the “hotspot.” Most patients presented with AI as the initial symptom followed by HH (or not); however, two cases presented with HH first, and further endocrine investigations confirmed AI. Among these variants, p.W39X was identified in two kindreds; the proband reported by M. Guclu et al. (17) presented with delayed puberty and small size of testes as the initial symptoms, whereas the other proband, reported by M. L. Raffin-Sanson et al., presented with fatigue, sore throat, and dizziness, which are consistent with AI (6). Moreover, the p.Ser259Pro mutation was also found in two families (11, 18), wherein both probands presented with AI initially, however, hypogonadism was absent in the case reported by C. M. Oh et al. (18). The reproductive phenotypes were variable in these patients, ranging from azoospermia to spontaneous fertility, even within the same family, as shown by M. C. C. Vargas et al. (20), referring to a family with spontaneous fertility and a variable spectrum of reproductive phenotypes, in which all males presented a distinct degree of testosterone deficiency and fertility phenotypes, ranging from a variable degree of hypogonadism, oligoasthenoteratozoospermia to spontaneous fertility. Additionally, the same variant can cause diverse phenotypes within one kindred as reported in a family with the p.Trp39X variant; the proband was diagnosed with AI at the age of 19 years but exhibited a preserved hypothalamic–pituitary–gonadal axis; in contrast, his nephew experienced AI crisis at the age of 2 weeks (6). The underlying mechanism remains obscure. The mutation by itself as well as the repressive role of *NR0B1* in steroidogenic factor-1 (SF1/Ad4BP, NR5A1) may be involved in this mechanism. DAX1 is expressed in tissues involved in steroid hormone production and reproductive function (21). This expression pattern overlaps significantly with that of another nuclear receptor, SF-1 (22). Previous studies have suggested that DAX1 is a negative regulator that represses SF-1-mediated transactivation of various genes involved in steroidogenesis (23). The loss of this inhibitory property in the LBD of DAX1 in the *NR0B1* variant has been demonstrated to be responsible for the pathology of X-linked AHC and HH (24–30).

In addition, the proband’s serum triglyceride (TG) level was significantly high, which can be related to a low testosterone level. The plausible mechanism for the significant association between TG and testosterone levels in men is that low testosterone levels promote insulin resistance (31) by predisposing to visceral obesity, leading to a dysregulation of fatty acid metabolism, which in turn promotes insulin resistance (32). As reported previously, the relative risk of death in Swedish patients with AD is more than two-fold higher than that in the background population, primarily because of cardiovascular disease (CVD); elevated triglycerides are one of the CVD risk factors (33). Combining the dietary habits as well as no family history of hyperlipidemia makes it difficult to explain the common diseases of lipid metabolism. Therefore, whether or not the patient has a deletion of the glycerol kinase (GK) gene should be considered. The GK gene is located in Xp21.3, in a critical region of approximately 50–250 kb, close to the genomic coordinate of the AHC gene, and located distal to the Duchenne muscular dystrophy gene (34). Genomic analysis confirmed that the GK gene was unaffected, excluding combined glycerol kinase deficiency. Moreover, the serum VLCFA level was normal; therefore, adrenoleukodystrophy (ALD), another X-linked AI disorder, was also excluded. ALD is associated with primary testicular failure and characterized by primary AI and demyelination of the central or peripheral nervous system. The disease results from impaired transport of VLCFA into peroxisomes for beta-oxidation, which results in elevated levels of VLCFA in plasma and tissues (35).

## Conclusion

In summary, we identified a novel frameshift variant of *NROB1*. This finding is important for expanding our knowledge of the phenotype and genotype of X-linked AHC. Functional studies of AHC patients are necessary to elucidate the biological effects of the identified mutation on the development and function of the adrenal gland and the hypothalamic–pituitary–gonadal axis.

## Data Availability Statement

The datasets presented in this study can be found in online repositories. The names of the repository/repositories and accession number(s) can be found below: https://ngdc.cncb.ac.cn/, HRA002312.

## Ethics Statement

Ethical review and approval were not required for the study on human participants in accordance with the local legislation and institutional requirements. Written informed consent was obtained from the patient prior to his inclusion in the study.

## Author Contributions

JX designed and led this study. FZ and MZ searched the literature and drafted the manuscript. XD collected patient information, recruited the patient. YL analyzed the data. JX revised the manuscript. All authors read and approved the final manuscript.

## Funding

This work was supported by grants from the National Natural Science Foundation of China (No. 81770736).

## Conflict of Interest

The authors declare that the research was conducted in the absence of any commercial or financial relationships that could be construed as a potential conflict of interest.

## Publisher’s Note

All claims expressed in this article are solely those of the authors and do not necessarily represent those of their affiliated organizations, or those of the publisher, the editors and the reviewers. Any product that may be evaluated in this article, or claim that may be made by its manufacturer, is not guaranteed or endorsed by the publisher.

## Abbreviations

ACTH, Adrenocorticotropic Hormone; AHC, Adrenal Hypoplasia Congenita; CAH, Congenital Adrenal Hyperplasia; DAX1, Dosage-sensitive sex reversal, adrenal hypoplasia congenital critical region on the X chromosome, gene 1; GnRH, Gonadotropin releasing Hormone; LH, luteinizing hormone; FSH, follicle-stimulating hormone; hCG, human chorionic gonadotropin; VLCFA, very long chain fatty acid, 17-OH, hydroxyprogesterone; CT, computed tomography; HH, hypogonadotropic hypogonadism.
