# Supplementary material for: Case Report: A Novel Truncating Variant of NR0B1 Presented With X-Linked Late-Onset Adrenal Hypoplasia Congenita With Hypogonadotropic Hypogonadism
Source: Front Endocrinol (Lausanne). 2022 Jun 16;13:897069. doi: 10.3389/fendo.2022.897069 (PMC9243302; doi:10.3389/fendo.2022.897069)
Supplement: Supplementary file 1 [file Table_1.docx]

| **Supplementary Table1 Summary of clinical cases of adult-onset adrenal insufficiency and/or hypogonadotrophic hypogonadism associated with mutations of the DAX-1 (NR0B1) gene** | | | | | | | | |  |
| --- | --- | --- | --- | --- | --- | --- | --- | --- | --- |
| Genotype | Onset age (years) | Initial symptoms | Other symptoms | Diagnosis examination | Family members affected | Treatments | Follow-up | References |  |
| Missense p.Ile439Ser | 28 | AI (fatigue) | HH (impaired libido and infrequent erections,6 mL testes) | ↓Na+, ↑K+ ↓peak cortisol to SST, ↑ACTH, ↓renin, undetected aldosterone  HPG axis:↓testosterone | Mother：  Phenotype：healthy  Genotype: heterozygous | Hydrocortisone (35 mg/d), fludrocortisone (100 μg/d)  Testosterone enanthate (250 mg intramuscularly every 3 weeks) | No improvement in semen analysis after 10 months treatment with gonadotropin | (1) |  |
| Missense p.Tyr380Asp | 28 | HH ( underdeveloped secondary sexual characteristics,5ml testes ) | none (compensated AI) | ↑ACTH, ↓peak cortisol to SST, normal renin, small adrenal glands  HPG axis: azoospermia,↓testosterone, | Mother:   Phenotype：healthy  Genotype: heterozygous | Cortisone acetate (25 mg total daily) for 2 years  Gonadotropins replacement | No improvement in semen analysis after 8 months treatment with gonadotropin | (2) |  |
| Nonsense p.Gln37X | 20 | AI ( fatigue, nausea, and hyperpigmentation) | HH (4 ml testes) | ↓cortisol, ↑ACTH，↓renin，↓aldosterone   HPG axis: azoospermia, ↓testosterone, ↓inhibin | _ | Gonadotropins replacement | No improvement in semen analysis after 6 months treatment with gonadotropin | (3) |  |
| p.Gln305Hisfs*67 (Deletion 305delG) | 17 | AI (skin pigmentation) | HH (18y, small testes) | ↓peak cortisol to SST, ↑ACTH, ↑PRA,↓renin atrophy of the adrenal glands  HPG axis: azoospermia,↓testosterone，↓LH and FSH | Mother:   Phenotype：healthy  Genotype: heterozygous  Brother :   Phenotype:AI age 5 years, HH age 21 years  Genotype: hemizygous | Replacement with hydrocortisone (30 mg)  Weekly injections of HCG (6000 IU) since 20y | 0 sperm count after treatment with gonadotropin since the age of 20 until age of 30 | (4) |  |
| Nonsense p.Trp39X | 22 | HH (delayed puberty, 2–3 ml testes) | AI (weak and dizzy) | ↓Na+, ↑K+, ↑ACTH, ↓peak cortisol to SST  HPG axis: ↓testosterone, ↓LH, normal FSH, | Brother:  Phenotype: 18y, HH, ↑ACTH, ↓peak cortisol to SST  Genotype: NA (died suddenly during intensive physical activity) | Glucocorticoid, mineralocorticoid replacement  Testosterone replacement | - | (5) |  |
| Nonsense p.Trp39X | 19 | AI (fatigue, sore throat, dizziness) | Normal testes volumes | ↓peak cortisol to SST, ↑ACTH, bilateral adrenal atrophy  HPG axis: oligospermia, normal LH/FSH/inhibin B levels | Mother:  Phenotype :healthy  Genotype: heterozygous    Sister:   Phenotype: healthy  Genotype: heterozygous   Nephew:   Phenotype: AI crisis age 2 weeks  Genotype: hemizygous   Brother:   Phenotype: age 36 years, compensated AI(↓peak cortisol to SST, ↑ACTH, ↓aldosterone) ↓testosterone, oligospermia  Genotype: hemizygous | Hydrocortisone 30 mg/day and 9a-fluorocortisone 100 mg/day  Without hormonal treatment for normal testosterone and gonadotropin levels | Age 33 years, fathered a first healthy son by IVF.  Age 35 years, a second healthy son by spontaneous conception. | (6) |  |
| Missense p.Ser259Pro | 28 | AI (presentation not mentioned) | NONE | ↓cortisol, ↑ACTH, adrenal hypoplasia  HPG axis: normal testosterone | Brother:   Phenotype: AI age 36 years, testosterone not done   Genotype: Missense p.S259P   Mother:   Phenotype: healthy,  Genotype: Missense p.S259P    Maternal cousin:   Phenotype: primary AI  Genotype: NA | - | Normal | (7) |  |
| Missense p.Ser259Pro | 19 | AI (skin pigmentation) | HH (Age 38 years, low libido) | ↓Na+,↑K+, ↓peak cortisol to SST  HPG axis: ↓testosterone, normal LH, ↑FSH | Brother:   Phenotype: AI, no children  Genotype: NA | Hydrocortisone and fludrocortisone replacement  testosterone replacement | No family plan | (8) |  |
| Missense p.Pro279Leu | 30 | AI (hyperpigmentation) | Age 37 years, HH, ejaculatory failure and subfertility, small testes | ↓peak cortisol to SST, ↓aldosterone   HPG axis:↓ testosterone, ↓LH, normal FSH, azoospermia | - | hydrocortisone and fludrocortisone  hCG and hMG therapy, androgen replacement | Remained azoospermia after gonadotropin therapy. Failed on TESE. Androgen replacement thereafter  A healthy child following assisted conception via donor insemination | (8) |  |
| Missense p.Leu386Phe | 17 | AI (hyperpigmentation) | Not yet developed secondary sexual characteristics, 4ml testes | ↓cortisol, ↑ACTH, adrenal hypoplasia, small pituitary gland  HPG axis: ↓testosterone, ↓FSH, ↓LH, | - | - | - | (9) |  |
| Missense p.Tyr378Cys | 41 | AI (progressive weight loss, salt craving, and cutaneous hyperpigmentation) | Age 25 years, erectile dysfunction and decreased libido, 3ml testes | important volume reduction in the adrenal glands  HPG axis: ↓testosterone, ↓FSH, normal LH, undetectable inhibin | Maternal uncle:  Phenotype: fathered a healthy son at 39 y; azoospermia, hypogonadism symptoms at 58y; PAI at 64y.  Genotype: hemizygous  Brother:   Phenotype: fathered a healthy son at 32 y; PAI, severe oligospermia, asthenospermia, teratospermia at 36y.  Genotype: hemizygous | - | No children | (10) |  |
| p.Pro345Argfs*27 | 17 | AI (skin hyperpigmentation) | HH (26y, delayed puberty, absence of secondary sexual characteristics) | ↓Na,↓cortisol, ↑ACTH, adrenal hypoplasia  HPG axis: ↓testosterone, normal FSH, normal LH, | Maternal uncle:  Phenotype: Addison’s disease at 30y  Genotype: Hemizygous  The other maternal uncle:  Phenotype: Addison’s disease at 30y, died of adrenal crisis at 35y  Genotype: NA | hydrocortisone (15-35 mg/d) and fludrocortisone replacement  androgen replacement | gave up on family plans |  |  |
| AI: adrenal insufficiency, HH: hypogonadotrophic hypogonadism, IVF: in vitro fertilization, SST: short synacthen test, TESE: testicular sperm extraction, NA: not available | | | | | | | |  |  |
|  |  |  |  |  |  |  |  |  |  |

1. Tabarin A, Achermann JC, Recan D, Bex V, Bertagna X, Christin-Maitre S, et al. A novel mutation in DAX1 causes delayed-onset adrenal insufficiency and incomplete hypogonadotropic hypogonadism. J Clin Invest. 2000;105(3):321-8.

2. Mantovani G, Ozisik G, Achermann JC, Romoli R, Borretta G, Persani L, et al. Hypogonadotropic hypogonadism as a presenting feature of late-onset X-linked adrenal hypoplasia congenita. J Clin Endocrinol Metab. 2002;87(1):44-8.

3. Ozisik G, Mantovani G, Achermann JC, Persani L, Spada A, Weiss J, et al. An alternate translation initiation site circumvents an amino-terminal DAX1 nonsense mutation leading to a mild form of X-linked adrenal hypoplasia congenita. J Clin Endocrinol Metab. 2003;88(1):417-23.

4. Sekiguchi Y, Hara Y, Matsuoka H, Hayashi Y, Katsumata N, Hirata Y. Sibling cases of Addison's disease caused by DAX-1 gene mutations. Intern Med. 2007;46(1):35-9.

5. Guclu M, Lin L, Erturk E, Achermann JC, Cangul H. Puberty, stress, and sudden death. Lancet. 2010;376(9751):1512.

6. Raffin-Sanson ML, Oudet B, Salenave S, Brailly-Tabard S, Pehuet M, Christin-Maitre S, et al. A man with a DAX1/NR0B1 mutation, normal puberty, and an intact hypothalamic-pituitary-gonadal axis but deteriorating oligospermia during long-term follow-up. Eur J Endocrinol. 2013;168(4):K45-50.

7. Oh CM, Chun S, Lee JE, Lee JS, Park S, Gee HY, et al. A novel missense mutation in NR0B1 causes delayed-onset primary adrenal insufficiency in adults. Clin Genet. 2017;92(3):344-6.

8. Kyriakakis N, Shonibare T, Kyaw-Tun J, Lynch J, Lagos CF, Achermann JC, et al. Late-onset X-linked adrenal hypoplasia (DAX-1, NR0B1): two new adult-onset cases from a single center. Pituitary. 2017;20(5):585-93.

9. Suthiworachai C, Tammachote R, Srichomthong C, Ittiwut R, Suphapeetiporn K, Sahakitrungruang T, et al. Identification and Functional Analysis of Six DAX1 Mutations in Patients With X-Linked Adrenal Hypoplasia Congenita. J Endocr Soc. 2019;3(1):171-80.

10. Vargas MCC, Moura FS, Elias CP, Carvalho SR, Rassi N, Kunii IS, et al. Spontaneous fertility and variable spectrum of reproductive phenotype in a family with adult-onset X-linked adrenal insufficiency harboring a novel DAX-1/NR0B1 mutation. BMC Endocr Disord. 2020;20(1):21.
